# Recurrent optic neuritis in a patient with Sjogren syndrome and neuromyelitis optica spectrum disorder: A case report

**DOI:** 10.1097/MD.0000000000023029

**Published:** 2020-11-06

**Authors:** Wei Zheng, Xiaoping Liu, Xiujuan Hou, Yuelan Zhu, Taotao Zhang, Liang Liao

**Affiliations:** a Beijing University of Chinese Medicine, Beijing; b Department of Endocrinology, The First Affiliated Hospital of Zhejiang Chinese Medical University, Hangzhou, Zhejiang; c Department of Rheumatology; d Department of Ophthalmology, Dongfang Hospital Beijing University of Chinese Medicine, Beijing, China.

**Keywords:** anti-aquaporin4 antibody, neuromyelitis optica spectrum disorder, Sjogren syndrome

## Abstract

**Rationale:**

Neuromyelitis optica spectrum disorder (NMOSD) patients, especially those with anti-aquaporin-4 antibody positivity, a water channel expressed on astrocytes, is often accompanied by autoimmune diseases (ADs) including Sjogren syndrome (SS). Here, we report a case of a young Chinese woman with recurrent optic neuritis who was diagnosed with asymptomatic SS and NMOSD.

**Patient concerns:**

A 22-year-old Chinese woman suffered from optic neuritis for 3 years. The main manifestation was recurrent loss of vision. The anti-aquaporin-4 antibody was positive in the cerebrospinal fluid, and she was diagnosed with NMOSD. Other laboratory tests revealed positive anti-SSA and anti-SSB antibodies, and labial gland biopsy showed lymphocytic infiltration. She also fulfilled the international criteria for SS.

**Diagnosis:**

On the basis of recurrent vision loss and laboratory examination, we defined the patient with SS accompanied by NMOSD.

**Interventions:**

When the patient first experienced vision loss, the corticosteroid treatment in the external hospital was effective, and her visual acuity improved significantly. However, in several later attacks, such treatment was no longer obviously effective. Considering the patient's condition, she was treated with corticosteroids, cyclophosphamide, and immunoglobulin therapy on admission.

**Outcomes:**

The patient's visual acuity was increased to the right eye 20/800 and left eye finger counting when she was discharged from the hospital.

**Lessons:**

SS accompanied with NMOSD is common in clinical practice, and always with the positive Anti-AQP4 antibody as a potential biomarker. Patients with SS and NMOSD showed significant neurological symptoms and had a worse prognosis than SS patients with negative anti-AQP4 antibody because of cross-immunity between anti-SSA antibody and anti-AQP4 antibody. Rheumatologists and ophthalmologists should pay attention to this and perform appropriate tests.

## 1 Introduction

Sjogren syndrome (SS) is classically defined as an autoimmune inflammatory disease, which affects all exocrine glands, especially lacrimal and salivary glands. As many as 50% of SS patients may experience extraglandular systemic manifestations.^[1]^

Optic neuritis (ON), a disease with various inflammatory lesions involving the optic nerve, is one of the most common blinding diseases among young and middle-aged people.^[2]^ It occurs independently or as the first symptom of neuromyelitis optica spectrum disorder (NMOSD). NMOSD is a group of antigen-antibody mediated central nervous system (CNS) inflammatory demyelinating disease spectrum, mainly involving the optic nerve and spinal cord. The clinical manifestations of NMOSD are diverse, including 6 groups of core clinical symptoms: ON, acute myelitis, medulla oblongata syndrome, acute brainstem syndrome, acute diencephalon syndrome, and brain syndrome.^[3]^ NMOSD is associated with autoimmune diseases (ADs), such as SS and systemic lupus erythematosus (SLE).^[4–6]^

Here, we report a young woman with “ON” as the first diagnosis. With the development of the disease, we finally diagnosed her as “SS” and “NMOSD.” The patient's condition was significantly improved after corticosteroid and immunosuppressive drug treatment.

## 2 Case report

In 2015, a 22-year-old Chinese woman suffered a sudden loss of vision in her right eye with eye movement pain. She was diagnosed with “ON” and recovered after high-dose corticosteroid therapy in a local hospital. In April 2016, her visual acuity of the left eye suddenly declined to 20/100, accompanied by pain when the eyeball rotated. The same therapy was administered, but visual acuity was only slightly improved. Furthermore, there was recurrence in the right eye once, and there was recurrence in the left eye twice in the following 2 years. Unfortunately, the effect of corticosteroids worsened and the visual acuity became very poor.

**Figure 1:**
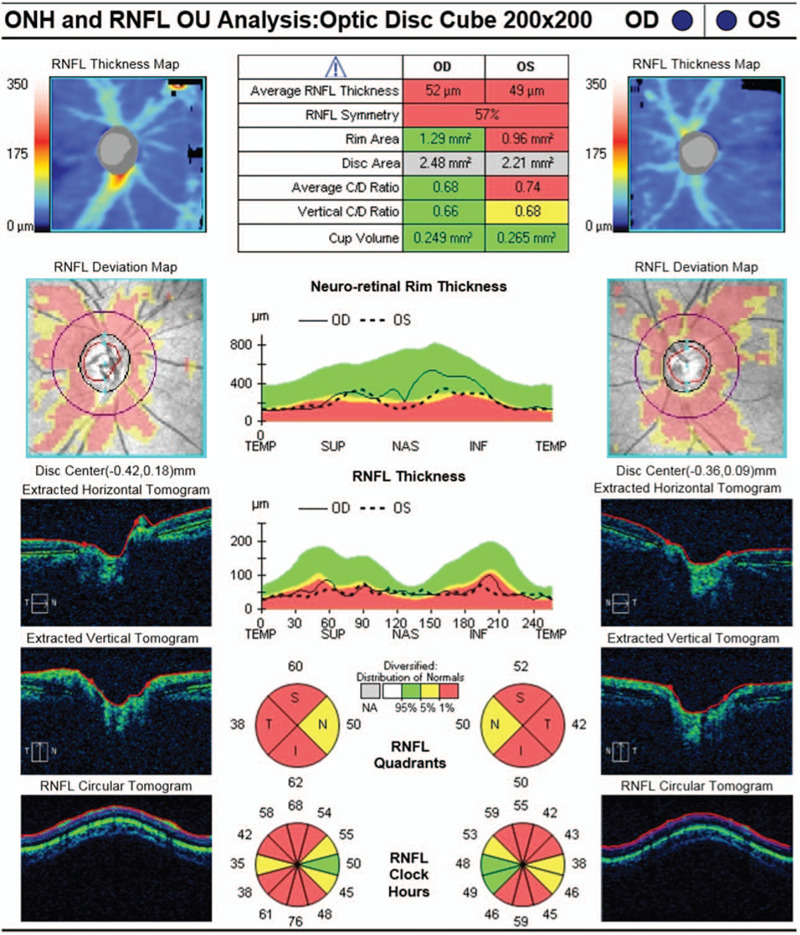
The retinal nerve fiber layer around the optic disc were significantly thinner than normal in the optic coherence tomography assay (HD-OCT, Cirrus 4000, Carl Zeiss).

In October 2018, when her left eye suffered again with visual acuity dropping to no light perception, corticosteroids did not work anymore. Finally, when there was recurrence in the right eye at the end of November 2018, visual acuity of both eyes dropped to no light perception. She then came to our ophthalmology clinic, with both eye blindness and pupil dilation; only her right eye had a mild light response. Fundus examination revealed pale binocular disc and miniature blood vessels. The patient had neither spastic weakness nor sensory signs of the legs and limbs. Visual evoked potentials examination indicated that the P100 waveform of both eyes nearly disappeared, while the retinal nerve fiber layer around the optic disc was significantly thinner than that of the normal in the optic coherence tomography assay (Fig. 1). Magnetic resonance imaging (MRI, with contrast) showed no longitudinally extensive myelitis signals at the thoracic or lumbar spine, and there were no demyelination-enhancing changes in the brain, but some suspicious signals existed at the top right of the frontal lobe, which was considered ischemic lesions. Laboratory examination showed that the anti-aquaporin 4-antibody was positive in the cerebrospinal fluid (CSF, cell transfection).

The patient fulfilled the 2015 international consensus diagnostic criteria for NMOSD.^[3]^ She was treated with intravenous methylprednisolone 0.5 g/day for 5 days, then reduced to 0.25 g/day for 3 days, followed by daily reduced prednisolone start with 50 mg oral administration.

**Figure 2:**
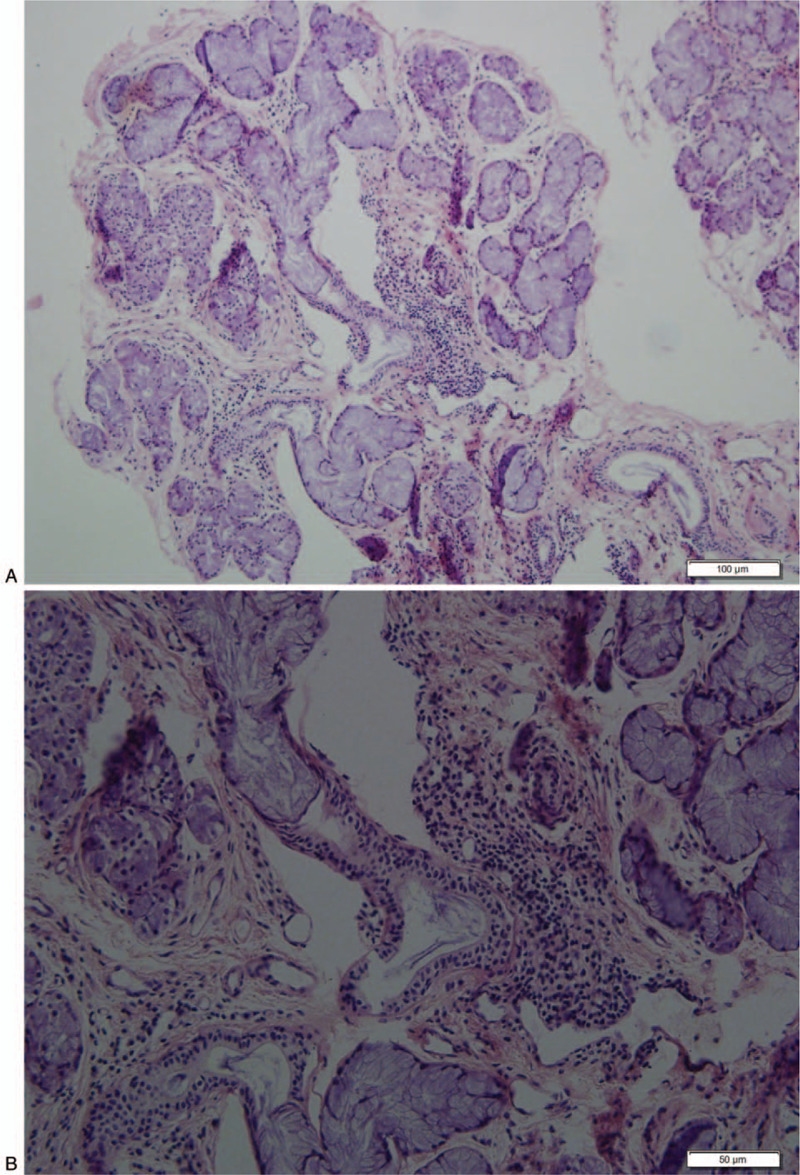
Minor salivary gland biopsy revealed chronic inflammation with small intralobular aggregates of plasma cells. Focus score was 2. Scale bar, 100 nm (A), 50 nm (B), respectively.

Further examinations showed that antibodies associated with SS were positive [anti-nuclear antibody (ANA) (titer, 1:1000), anti-SSA antibody, anti-SSB antibody, anti-Ro-52 antibody]. Complement C3: 0.77 g/L. Labial gland biopsy showed partial acinar atrophy of the salivary gland tissue and focal aggregation of interstitial lymphocytes (2 foci, > 50 lymphocytes per foci) (Fig. 2). The rheumatoid factor (RF), erythrocyte sedimentation rate (ESR), and C-reactive protein (CRP) were negative. However, there were no common symptoms of SS, such as dry mouth and eyes. Dry eye examinations were performed as follows: Schirmer - I (Sch- I) test results showed 4 mm for both eyes, tear breakup time (TBUT) was 3 s for the right eye and 6 s for the left eye, and the tear meniscus height was 0.11 mm for the right eye and 0.15 mm for the left eye. Although the patient did not have any obvious dry eye symptoms, she fulfilled the 2016 American College of Rheumatology (ACR)/The European League Against Rheumatism (EULAR) Classification Criteria of SS. Hence, she was diagnosed as “1. NMOSD; 2. SS.” On the basis of corticosteroid treatment, she was administered cyclophosphamide (CTX 0.4 g/day) intravenous drip once per week for 2 weeks and intravenous immunoglobulin (17.5 mg/kg/day) daily for 5 days. Her visual acuity returned to the binocular hand move after 5 days of treatment. Four weeks later, the patient’ s visual acuity increased to 20/800 in the right eye and finger counting in the left eye.

## 3 Discussion

SS is a systemic autoimmune inflammatory disease. Approximately 20% of SS have neurological manifestations, including peripheral nervous system damage and/or CNS damage.^[7]^ In 25% of cases, CNS damage may be the first manifestation.^[8]^ CNS manifestations are heterogeneous, including recurrent ON, focal neurological deficit symptoms, diffuse brain damage symptoms, dyskinesia, or spinal cord lesions.

NMOSD is a rare ophthalmic disease; its typical clinical symptoms are ON and recurrent longitudinally extensive transverse myelitis. NMOSD incidence is 0.05 per 100,000 among populations in Abu Dhabi.^[9]^ A retrospective study revealed that aquaporin 4 (AQP4)-positive patients are closely associated with nonorgan-specific autoantibodies, including anti-SSA, anti-SSB, anti-ANA, and anti-dsDNA antibodies.^[10]^ Previous studies have shown that SS and SLE are related to NMOSD in systemic ADs.^[7]^ The existence of SS in patients with NMOSD has been estimated to be 2% to 30%.^[11]^ The majority of AQP4-IgG seropositive patients (NMOSD-related biomarker) are associated with a high intensity of inflammatory disease activity. Those patients have been demonstrated to have a higher titer of anti-SSA/SSB antibodies and a more acute course of SS.

In this case report, the patient had a history of recurrent ON, and the AQP4 in CSF was positive. MRI showed no longitudinally extensive myelitis signals at the thoracic or lumbar spine and only some ischemic lesions in the right frontal lobe. Hence, she was diagnosed with NMOSD. However, no immune-related examination was performed in the early stage of the disease, and it was difficult to determine whether SS appeared at the beginning of the disease or not. Furthermore, we questioned the patient's past medical history, no common symptoms, such as dry mouth or dry eyes, while the SS-related diagnoses were positive, including ANA, anti-SSA antibody, anti-SSB antibody, anti-Ro-52 antibody, Sch-I test, TBUT, and labial gland biopsy. Although the main manifestation of this patient was optic nerve damage, this phenomenon can define that the patient was also suffering from SS according to the SS classification criteria that ACR/EULAR issued in 2016. It has been found that 33% of SS patients have no typical SS symptoms, while CNS damage is clearly involved, but dry mouth, dry eyes, and other symptoms will appear successively in the next 5 years.^[12]^ This is consistent with the situation described in this case report.

Aquaporins (AQPs) are a family of 13 small proteins expressed at plasma membranes in many types of cells that transport water and some small solutes such as glycerol to across cell membranes. They are widely expressed in the body, mainly in cells that are involved in fluid transport such as epithelial cells.^[13]^ Some AQPs have been detected in lacrimal and salivary glands, AQP5 is one of them and has an important role in saliva and tear secretion.^[14]^ AQP5 is diminished in lacrimal glands of SS patients.^[15]^ Anti-AQP4, due to its pathogenic and diagnostic role in NMOSD, is the most well-known AQP antibody.^[13]^ AQP4-IgG can penetrate the blood--brain barrier (BBB) and react with AQP4 in astrocyte feet, initiates an immune response, which mediates demyelinating lesions, inflammatory cell infiltration, and hyaline degeneration of blood vessels. Some researchers considered that the coassociated mechanisms of SS and NMOSD may be related to the AQP epitope diffusion hypothesis.^[16]^ AQP4 and AQP5 are structurally homologous, 50% of the gene sequence of AQP4 and AQP5 is common.^[17]^ There is likely to be a common target, resulting in anti-AQP4 antibody binding to both AQP4 and AQP5 in multiple sites of exocrine glands, brain, and other sites, and the subsequent reactions may lead to the coexistence of SS and NMOSD, although the internal mechanism between SS and NMOSD remains to be elucidated.

Further, some immune-mediated inflammatory reactions can destroy the BBB, which is conducive to AQP4 entering the CNS.^[18]^ SSA antigen is present in vascular endothelial cells, and anti-SSA antibody is thought to cause endothelial damage, which is associated with disruption of the BBB. The presence of SSA antigen in SS patients complicated with NMOSD may make the condition worse, because more AQP4 will enter into CNS through the destroyed BBB. In a Chinese population-based study, white blood cells and proteins in the CSF, C-reaction protein, and immunoglobulin G in serum were higher in NMOSD with AD than in NMOSD without AD, which indicated that the immune status of NMOSD with AD is more active.^[19]^

The treatment of SS combined with NMOSD is based on experience in the treatment of ADs and/or NMOSD. High-dose corticosteroid pulse therapy is recommended as first-line treatment in the acute phase. Plasma exchange, immunoglobulin, and cyclophosphamide can be considered when the curative effect of corticosteroids is poor or the disease occurs repeatedly. Sequential treatment can choose immunosuppressant drugs, such as azathioprine (AZA; 2.5–3 mg/kg), mycophenolate mofetil (MMF; 2–3 grams/day), and/or biological drugs.^[16,20]^ In this patient with SS and NMOSD, recurrent visual loss could not be effectively controlled by corticosteroid therapy only. We then adopted the combination therapy scheme with corticosteroids, cyclophosphamide, and immunoglobulin. After 2 months of treatment, the patient's vision improved significantly.

## Acknowledgments

The authors thank the patient for her consent to publish her case and the related pictures. The authors thank the National Natural Science Foundation of China (81973909, 81874438) for their support.

## Author contributions

**Conceptualization:** Xiaoping Liu.

**Funding acquisition:** Yuelan Zhu

**Investigation:** Taotao Zhang

**Resources:** Liang Liao

**Supervision:** Yuelan Zhu, Xiujuan Hou, Xiaoping Liu, Liang Liao.

**Writing – original draft:** Wei Zheng.

**Writing – review & editing:** Xiujuan Hou, Liang Liao, and Wei Zheng.
